# Early Decay of Teeth in Britain

**Published:** 1899-11

**Authors:** 


### Early Decay of Teeth in Britain.

This author calls attention to a condition of things which we had not supposed was so prevalent in Great Britain as in this country, but it appears that the children's teeth in Her Majesty's dominion are suffering more at the present time than was formerly the case. He believes that the cause is to be found in deficient and artificial feeding in infants. This causes abnormal development of the jaws, which is the source of nasal abnormalities, and the arched jaw, which is coming to be so common, is produced within the first twelve months of infant life by abnormal feeding. The early decay of the teeth is one of the symptoms of want of care of the infant. He calls for a commission of the British Medical Association to investigate the subject and to ask for the co-operation of the Americans, and probably also the Germans, French and Italians in this matter.—*James Cantlie, in British Medical Journal.*

